# Geotechnical Evaluation of Loess Modifications as the Sustainable Compacted Soil Liner Material in Solid Waste Landfill

**DOI:** 10.3390/ma15144982

**Published:** 2022-07-18

**Authors:** Zhengrui Zhang, Siti Jahara Matlan, Hao Wang, Ahad Amini Pishro, Lili Zhang, Xian Gao, Zhao Liang, Xiaoyi Liu, Peigen Zhao

**Affiliations:** 1 Civil Engineering Programme, Faculty of Engineering, University of Malaysia Sabah, Kota Kinabalu 88400, Sabah, Malaysia; zhangzhengrui@suse.edu.cn; 2 College of Civil Engineering, Sichuan University of Science & Engineering, Zigong 643000, China; ahad.ap@suse.edu.cn (A.A.P.); zhanglili@suse.edu.cn (L.Z.); gaoxian@suse.edu.cn (X.G.); 3 Gansu Province Urban And Rural Planning Design & Research Institute, Co., Ltd., Lanzhou 730070, China; wanghao20041723@163.com (H.W.); zhaopeigen3205@163.com (P.Z.); 4 College of Civil Engineering, Lanzhou Jiaotong University, Lanzhou 730070, China; 12201177@stu.lzjtu.edu.cn (Z.L.); 12201184@stu.lzjtu.edu.cn (X.L.)

**Keywords:** sustainable compacted soil liner, loess modifications, attapulgite-lime, attapulgite-cement, permeability coefficient, NMR-SEM-XRD, shear strength, Lanzhou China

## Abstract

This paper studied the anti-seepage ability of the modified loess by using attapulgite, which is abundant in local areas. The possibility of using the modified loess as the sustainable compacted soil liner material in a solid waste landfill was also considered in this research. The materials were then evaluated using Nuclear Magnetic Resonance (NMR), Scanning Electron Microscopy (SEM), X-ray Diffraction (XRD), and an Impermeability Mechanism (IM). The experimental results showed that the permeability coefficient of the attapulgite-modified loess decreases significantly with increasing attapulgite content. However, it becomes less significant when the attapulgite level approaches 10%. Both cases can meet the landfill impermeability requirements, based on the attapulgite content remaining at 10%, adding 15% lime or 5% cement, respectively. The triaxial consolidation and drainage experiment was carried out to investigate the shear strength of the materials under three different working circumstances. The stress–strain curves of each specimen were produced, as were the cohesion and internal friction angle values. This research lays the groundwork for using attapulgite-modified loess as a landfill lining material. It establishes a solid platform for future studies on attapulgite adsorption and purifying performance in landfills.

## 1. Introduction

With the acceleration of industrialization and modernization, people’s living standards are gradually improving, while the amount of urban waste generated is increasing. While humankind has promoted and benefited from technological progress, it has also paid a substantial environmental cost [1]. Suppose that scientific and safe disposal methods are not used, these solid wastes will not only waste a large amount of national land and seriously pollute the environment, but they will also spread germs, harm people’s physical and mental health, and impede the long-term development of China’s social economy [2]. A sanitary landfill is currently the most commonly used type of landfill. A sanitary landfill is a comprehensive treatment system that includes an anti-seepage layer, leachate collection, venting, and other features.

Lanzhou city, located in the loess plateau of northwest Gansu Province, China, is covered by a sizeable collapsible loess area [3]. The physical properties of loess are loose, and the pores between soil particles are large. The cementing material of silicate connects the particles. In the case of dry loess, its nature is more stable and has a specific bearing capacity. However, the gelling material between the particles is dissolved after water immersion. Under the influence of gravity, the particles will slide and sink [4,5]. As a result, in a dry environment, the carrying capacity is relatively strong. However, it will quickly form gullies, subsidence, and collapse under water erosion. So, the anti-seepage is particularly important when building a landfill in Lanzhou.

The two most commonly used lining materials in landfills are bentonite cushion and HDPE geomembrane. However, these two methods have advantages and disadvantages [6,7,8]. For bentonite cushion, it is widely used because of its excellent impermeable performance. The permeability coefficient can reach 5 × 10^−11^ cm/s under hydrostatic pressure of 1.0 MPa. Bentonite is a natural inorganic material that will not occur an aging reaction and will not cause any adverse impact on the environment. It is an environmentally friendly material. However, in the Lanzhou area, bentonite reserves are deficient. Suppose we choose this material in a landfill. In that case, it is needed to transport bentonite from a distant place, the construction cost will be very high [9], and the shear strength is also low. For HDPE geomembrane, it has excellent environmental stress cracking resistance, low-temperature resistance, aging resistance, corrosion resistance, a wide range of temperatures (−60°~+60°) a long service life (50 years). It is widely used in Domestic Garbage Landfill Seepage Prevention (DGLSP), Solid Waste Landfill Seepage Prevention (SWLSP), Sewage Treatment Plant Seepage Prevention (STPSP), Artificial Lake Seepage Prevention (ALSP), Tailings Treatment (TT), and other seepage prevention engineering. The anti-seepage effect is good, but the cost is high, and easy to be punctured or corroded. Based on the above analysis, these two materials are not particularly suitable for use in Lanzhou. Therefore, developing a low-cost and high-efficiency anti-seepage material is essential for the Lanzhou loess area. It will effectively solve the contradiction between the local garbage disposal and local economic backwardness, special Geology, and other factors.

Cement, lime, and bentonite are the most widely used materials to modify loess and reduce permeability and shear strength. Moein Ghadakpour et al. evaluated the effects of cement on the mechanical behavior of loess and clayey soil, and various laboratory tests were conducted [10]. Dmitri Steshenko et al. presented a new method for improving the weak structure of loess soils according to a design based on a reliability assessment of embankments on weak soils concerning the structural design [11]. They described a new complex soil compaction technology applied to collapsing soils [12]. Piotr Kanty conducted over 100 compressions and a dozen tension tests to study cement-fly ash-modified soil’s compression and tensile strength [13]. Zhang Zhengrui et al. [14] studied the lateral seepage control of roadbeds using a lime soil compaction pile and continuous wall. The experiment found modified loess using lime for seepage prevention on a deep collapsible loess embankment slope. They found that the lime pile and lime anti-seepage wall had a magnificent performance as lateral anti-seepage structures. The lime wall was better than the lime compacted pile.

Zhang Yuanhu et al. [15] studied the bentonite modified loess. They discovered that the permeability coefficient of bentonite modified loess met the specifications. NiuYaQiang et al. [16] found that using cement compaction piles reduces the lateral coefficient of permeability of a loess foundation. The research indicates that bentonite-modified loess can be used as a landfill liner material. However, rarely in the inventory of bentonite in the Lanzhou area. This requires long-distance transportation, which no doubt increases the construction cost. The above scholars conducted multiple studies on the modification of the loess. However, the primary purpose is to improve the bearing capacity of loess, and modified materials mainly are of the traditional materials, such as cement, lime, and bentonite. For the loess area, landfill anti-seepage research is relatively rare. Additionally, all the research did not combine Lanzhou city’s local geology, geomorphology, and economic conditions.

Attapulgite is a clay material with a large amount of storage in Gansu [17,18]; its particular adsorption function is widely used in industrial purification and adsorption fields [19]. Attapulgite-modified loess is a brand new, ecological, environmentally friendly material. It can prevent seepage, purify adsorption, and finally, provides double security for the landfill. However, the application of attapulgite to landfills is relatively rare. In this study, local attapulgite was used to modify local loess, its anti-seepage mechanism was studied, and its shear strength was tested. This study’s original contribution and innovation are that it used the attapulgite abundant in Lanzhou to modify the local loess, which provides solid economic support for the local development. Secondly, this study also provides a scientific basis for the next research on the adsorption of leachate in landfills by attapulgite-modified loess. This study is based on the local geological and economic factors.

## 2. Materials and Methods

### 2.1. Materials

The results of loess and attapulgite grain composition performed by the code “Standard for Geotechnical Test Methods” (GB/T 50123-2019) are presented in Table 1. The loess used in this study was collected from Lanzhou city. The attapulgite was purchased from the Zhangye Yuanjian attapulgite Technology Co., Ltd., Zhangye, China. The analysis revealed that the gradation of loess is not uniform and has a high permeability under natural conditions due to its large surface areas and pores within the soil particles. However, attapulgite has been ground and screened in the factory, hence the tiny particle size. The proportion of particle size between 0.05 mm and 0.01 mm is 67%. For loess, the liquid limit (LL) is 28.7%, the plastic limit (Pl) is 14.9%, and the plastic index is 13.8.

Table 2 shows the main chemical composition of loess and attapulgite. According to the data, loess and attapulgite all include a lot of SiO_2_, Fe_2_O_3_, and MgO. They also contain a small amount of CaO. These substances react with the calcium ion to produce calcium silicate material, increasing soil shear strength and reducing the permeability coefficient [20].

Cement can be used to modify loess because it is a good curing agent. After a series of physical and chemical reactions between cement and loess, the loess can harden into high-quality soil with integrity, water stability, and specific strength. As quicklime releases a lot of heat energy during digestion, this process accelerates the hardening of lime soil. In contrast, the permeability coefficient is relatively small [14]. P.C.32.5 composite Portland cement produced by Qilian Mountain Cement Group in Gansu province is used in this study. For lime, the main component is CaO, and the main component of cement is 2CaO·SiO_2_, 3CaO·SiO_2_, 3CaO·Al_2_O_3_, 4CaO·Al_2_O_3_·Fe_2_O_3_. The other property of cement is shown in Table 3.

### 2.2. Loess Modification Mixtures

At the same time, the pure loess specimen was also prepared to carry out the NMR, SEM, XRD, and Shear strength test and compared the results between the pure loess specimen and other group specimens. Table 4 shows the mix ratio of the specimens in the compaction test. Group 1 is the combination of loess and attapulgite. Group 2 combines loess, attapulgite, and lime when the attapulgite content remains unchanged at 10%. Group 3 combines loess, attapulgite, and cement when the attapulgite content remains unchanged at 10%.

### 2.3. Testing Methods

**Figure 1:**
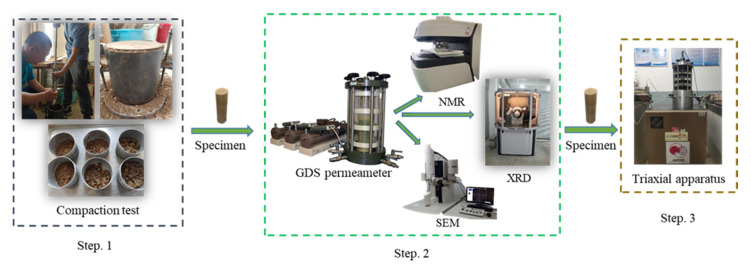
Experimental flow chart.

As the properties of lime or cement-modified soil have a great relationship with the curing age [16], the specimen with a curing period of 30 days was used in this experiment. Figure 1 is the experimental flow chart.

#### 2.3.1. Compaction Test

The first step is the compaction test. The maximum dry density and optimum water content are tested according to the mix ratio in Table 5. The method of spraying water and stirring mixes the soil sample evenly based on the water content measurements. After the samples were prepared, they were stored in a sealed plastic bag. After 48 h, it was used for a compaction test. The compaction cylinder has a 102 mm inner diameter, a volume of 947.4 cm^3^, and a weight of 2.5 kg for the hammer. The hammer’s bottom has a 51 mm diameter. The drop is 305 mm long and 592.2 kJ/m is the amount of energy. The compaction test method comprises three layers, each with 25 strokes, according to the GB/T50123-1999 Standard. Residual soil should not be higher than 5 cm in height.

#### 2.3.2. Permeation Test

After obtaining the max dry density and optional water content, we can make the standard penetration specimen and carry out the permeation test. When the permeability coefficient of porous material is less than or equal to 1.0 × 10^−7^ cm/s, the lateral wall leakage of the traditional rigid wall permeameter significantly impacts the permeability precision [15]. By contrast, the flexible wall permeameter has the advantages of accurate measurement and pressure permeation. In this test, the GDS-PERM flexible wall permeameter produced by GDS Company in Hook Hampshire, UK was used, referring to the ASTM D5084-03 standard. The permeability coefficient of modified loess was determined under constant head conditions. The size of the specimens is 50 cm in diameter and 100 cm high, with a cell pressure of 200 kPa, a back pressure of 40 kPa, and a base pressure of 20 kPa. Through the permeation test, we can determine the best ratio of each group that meets the requirement of the landfill construction code.

#### 2.3.3. NMR, SEM, and XRD Test

NMR (MacroMR12-150H-I, made in Shanghai, China), SEM (Zeiss Crossbeam 350/96, made in Berlin, Germany), and XRD (D8 ADVANCE, made in Berlin, Germany) tests were applied to analyze the principle of modified loess permeability coefficient reduction. NMR test will analyze the porn of the specimen. The principle of the NMR is to test the hydrogen atom of water in the sample and then convert it into porosity. Therefore, whether the sample is saturated or not will directly affect the accuracy of the test results. So, the sample must be fully saturated and carry out a B value check before the NMR test. SEM will observe the microstructure of the different mixtures of specimens, and SEM test subject to the specification of GB-T 18735-2002. XRD will analyze the composition of the specimen and subject it to the specification of GB-T 33502-2017. The above tests show that the reason for the reduction in the permeability coefficient will be illuminated from different angles.

#### 2.3.4. Shear Strength Test

Regarding the obtained optimal mix ratio, the specimens were remade for a static triaxial CD experiment to test the shear strength of the modified loess. Moreover, we determine the respective stress–strain curves, the cohesion (c) value, and friction angle (φ). Triaxial apparatus (GDS DYNTTS, made in Hook Hampshire, UK) will be used in this test. The shear test specimen is 80 cm high and 39.1 cm in diameter, with a shear rate of 0.012%/min until failure or strain reaches 20%, according to the GB/T50123-1999 Standard.

## 3. Results

### 3.1. Compaction Test

**Figure 2:**
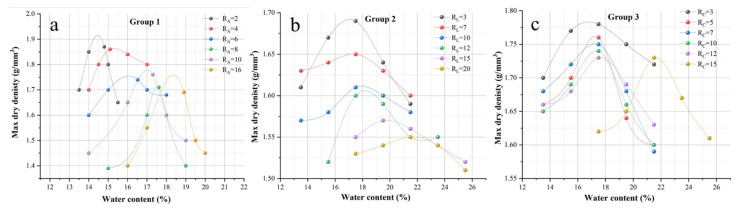
Compaction test results. (R—the percentage of material, A—attapulgite, L—lime, and C—cement). (**a**) Loess + Attapulgite, (**b**) Loess + Attapulgite + Lime, (**c**) Loess + Attapulgite + Cement.

Figure 2 shows the results of the compaction test. It can be seen from the figure that, in the three groups of experiments, with the increase in attapulgite, lime, and cement content, the optimal water content increases, and the maximum dry density decreases. Previous studies have shown that soil modification with cement, bentonite, volcanic ash, etc., increases soil water demand. Therefore, the optimal water content of the soil increases [21]. The curves in Figure 2 show the optimal water content and maximum dry density at each mixing ratio, as shown in Table 5. Since the density of attapulgite was smaller than that of loess, the maximum dry density of attapulgite decreased rapidly with the increase in attapulgite content in group 1. However, the larger surface area led to the continuous growth of optimal water content. Compared with group 1, the maximum dry density of groups (2) and (3) did not decrease significantly due to the density difference between lime, cement, and loess.

### 3.2. The Permeability Coefficient

**Figure 3:**
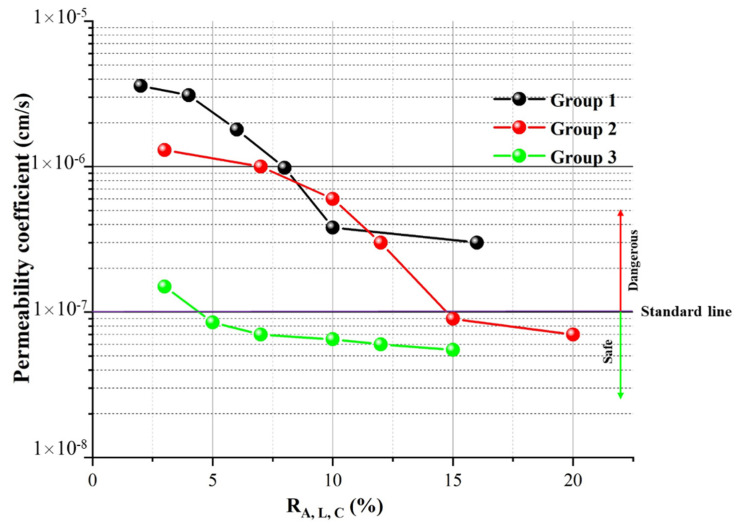
Permeation test results of three groups. (R—the percentage of material, A—attapulgite, L—lime, and C—cement).

Figure 3 shows the results of the infiltration experiment. For group 1, the permeability coefficient of the modified loess decreased significantly with the increase in attapulgite content. The curve slope was larger when the attapulgite content was less than 10%. When the attapulgite content was 10%, the permeability coefficient of the specimens dropped from 3.0 × 10^−6^ cm/s to 4.2 × 10^−7^ cm/s, decreasing by seven times. However, when the content was more than 10%, the curve flattened out, and the reducing rate of the permeability coefficient decreased. By the slope of the curve, it can be predicted that to meet the standard impermeability requirements (less than 1.0 × 10^−7^ cm/s) for group 1, the doping amount of attapulgite will reach 80% unscientific and uneconomical.

For group 2, adding lime based on attapulgite dosage was 10%. With the dosage increase in lime, the permeability coefficient of modified loess was also significantly reduced. When the lime content was 15%, the permeability coefficient reached the optimum specification requirements. With the content increase in lime, the permeability coefficient of the specimens continues to reduce the trends. For group 3, adding cement based on attapulgite dosage was 10%. With the increase in cement content, the coefficient of permeability of the specimens was reduced. Compared to the group 2 experiment, when the cement and lime dosage were all 3%, the permeability reduction rate of group 3 was 1.3 × 10^−7^ cm/s, group 2 of 1.2 × 10^−6^ cm/s, and the permeability reduction rate of group 3 was significantly higher than that of the group (1) and (2). Additionally, when the cement content was 5%, it had met the optimum permeability coefficient required by the specification. Therefore, it can be concluded that clay soil, lime, and cement can effectively reduce the permeability coefficient of loess [22]. Among the three groups of experiments, group 3 is the best in both anti-seepage effects. The amount of modified material, followed by group 2, group 1 has the worst impact, and the amount of modified material is higher.

### 3.3. NMR Test

**Figure 4:**
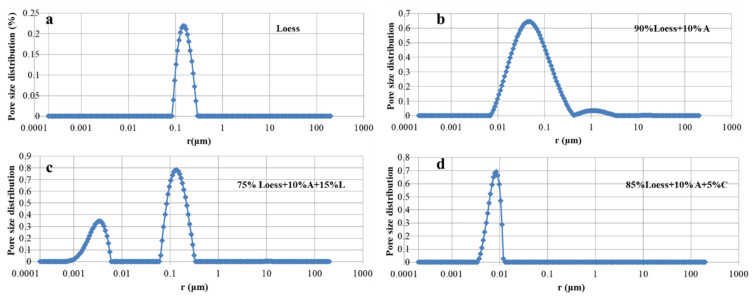
NMR test results. (A means attapulgite, L means lime, and C means cement). (**a**) Loess, (**b**) 90%Loess + 10%A, (**c**) 75%Loess + 10%A + 15%L, (**d**) 85%Loess + 10%A + 5%C.

Figure 4 shows pure loess’s NMR scanning results and three experimental specimen groups. Figure 4a is the scanning result of pure loess. The *X*-axis is the pore size, and *Y*-axis is the pore size distribution. It can be seen from the figure that the main pore size distribution of loess specimens is between 0.1 and 0.5 μm. Figure 4b is the test result of the specimen with 90% loess and 10% attapulgite in group 1. It can be seen from the figure that with the addition of 10% attapulgite, the pore size of the specimen shrinks, with an interval distribution of 0.008 μm–0.5 μm. Compared with the pore size of pure loess, the minimum pore size shrinks from 0.1 μm to 0.008 μm. Since the pores of loess are large, and the grains of attapulgite is small after they are mixed, the grains of attapulgite fill the pores between the loess, making the pores of the specimen smaller and reducing the permeability coefficient. Figure 4c shows the NMR scanning results of the specimen with 75% loess and 10% and 15% attapulgite and lime content in the group 2 experiments. It can be seen from Figure 4c that with the addition of 15% lime, pore sizes are mainly distributed in two ranges of 0.08 μm–0.4 μm and 0.001 μm–0.008 μm. Compared with Figure 4b, after adding 15% lime, the pores of the specimen became smaller, and the permeability coefficient decreased. Figure 4d shows the NMR scanning results of the specimen with 75% loess and 10% and 5% attapulgite and cement in the group 3 experiment. It can be seen from Figure 4c that, compared with Figure 4b, with the addition of 5% cement, the maximum pore size decreases from 0.5 μm to 0.02 μm, and the minimum pore size changes from 0.008 μm to 0.005 μm. The reduction in pore size means that the diameter of the percolation pore channel becomes smaller. According to Darcy’s law, the permeability coefficient of small pore size specimens will also decrease.

Combined with the penetration test result in Figure 3, we can conclude that the pore’s size is the main factor that directly affects the value of the permeability coefficient, and after attapulgite, lime, and cement mixed, the permeability coefficient of the specimen decreased significantly. Additionally, it can be seen from Figure 4 that after adding cement to the specimen, the porosity is the smallest. This result is consistent with the permeation test results in Figure 3.

**Figure 5:**
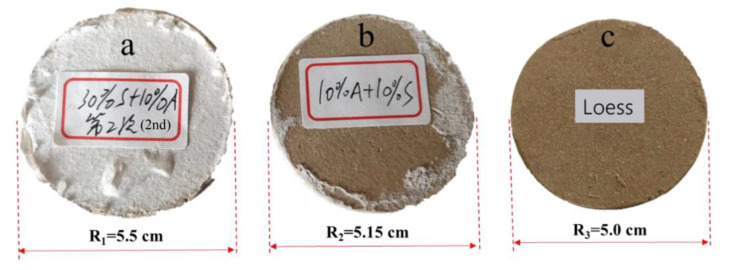
Change of specimen section diameter. (**a**) Attapulgite-Lime modified loess, (**b**) Attapulgite-Cement modified loess, (**c**) Loess.

Figure 5 shows the change in the cross-section area of the specimen after the NMR test. Since the principle of the NMR test is to detect hydrogen and then calculate the porosity, the specimen must be saturated before the test. The saturation of the specimen will directly affect the accuracy of the test, so the B value check should be tested before the test. After vacuum saturation of the specimen, an NMR test was carried out. In this dry-wet cycle, the volume of the specimen changed. As shown in Figure 5a, the volume expansion of the specimen with lime was evident. The specimen diameter changed from 5 to 5.5 cm, while the volume change of the specimen with cement was not noticeable. This is because the main component of lime is CaO, a chemical reaction that occurs when it meets water. This exothermic reaction produces a large amount of gas, which expands the specimen’s volume [23]. The reaction equation is as follows:

CaO + H_2_O = Ca (OH)_2_

In this process, the volume of the modified loess expands during the exothermic process. The gas constrained by the radial pressure in the permeability test cannot be released, making the internal pores of the specimen filled with gas. This effect also effectively blocks the seepage of water, which explains the reduction in the permeability coefficient of lime-modified loess.

### 3.4. SEM Test

**Figure 6:**
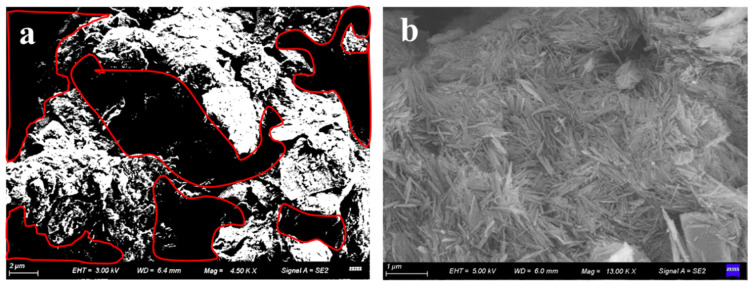
SEM test results of loess and attapulgite: (**a**) loess; (**b**) attapulgite.

Figure 6 shows the SEM results of loess and attapulgite. Figure 6a shows the results of loess. After the loess particles are magnified 4500 times, it can be seen that there are a large number of pores between the loess particles. Additionally, the particles are mainly connected from point to point. After water enters the loess, the junction’s cementitious material will dissolve. The particles will settle and become dense under the action of gravity [18]. Figure 6b shows the SEM results of attapulgite. It can be seen from the figure that the microstructure of attapulgite is needle shaped, with a relatively dense particle arrangement and few pores. Attapulgite has a large surface area and a unique crystal structure, making it particularly adsorptive.

**Figure 7:**
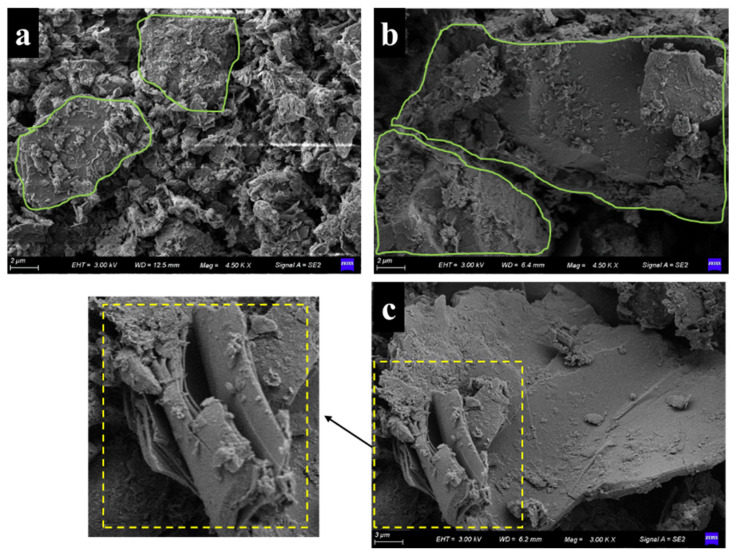
SEM test results of modified loess: (**a**) attapulgite-modified loess, (**b**) attapulgite-lime-modified loess, and (**c**) attapulgite-cement-modified loess.

Figure 7 shows the SEM results of modified loess. Figure 7a is the microstructure of attapulgite-modified loess. As shown in Figure 7a, under the same magnification, the loess particles of space structure have changed significantly after the intervention of attapulgite in loess. The loess particles become denser, pores are reduced, and the massive structure of a large area appears.

Moreover, attapulgite has a needle-like structure, resulting in a reinforcement effect when combined with loess, making them a flake whole. Figure 7b,c shows the microstructure of attapulgite-lime-modified loess and attapulgite-cement-modified loess, respectively. It can be seen from the figure that after adding lime and cement, loess particles coalesced into larger structures, especially after adding cement, and the effect was most apparent. Moreover, Figure 7c shows that the loess surface modified by cement attapulgite is smoother and flat. In the region of the yellow amplification, it can be seen clearly that the block structure is composed of the multilayer. According to previous studies, the cement-modified loess did not appear as a layered structure [21]. This is because the attapulgite mixed with loess formed a lamellar structure. After joining the cement-based on attapulgite-modified loess, cement cemented between lamellar structures, creating an overall multilayer structure. It can be inferred that the structure has higher strength, which is confirmed in the later triaxial shear experiments.

### 3.5. XRD Test

**Figure 8:**
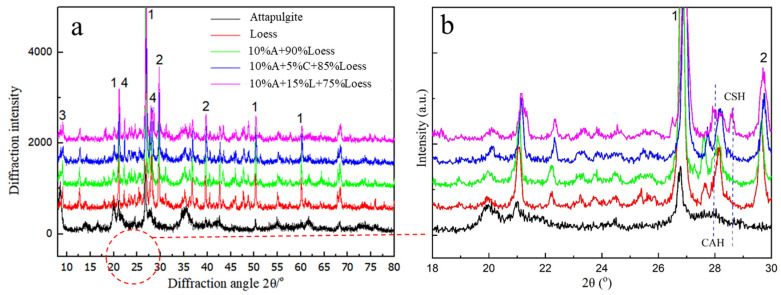
XRD test results of modified loess. (**a**) The XRD test results of loess and modified loess, (**b**) Enlarged view of XRD test results between 18° and 30°. (1, quartz; 2, calcite; 3, clay minerals; 4, feldspar, 5, palygorskite. A, attapulgite, L, lime, C, cement).

Figure 8 shows the XRD test results of loess, attapulgite, attapulgite-modified loess, attapulgite-lime-modified loess, and attapulgite-cement-modified loess. The loess is mainly composed of quartz, calcite, clay minerals, and feldspar. The main mineral of attapulgite is palygorskite. In addition to palygorskite, there is a lot of α-quartz (α-SiO_2_) diffraction peaks, which should be associated with quartz impurities. The diffraction pattern of attapulgite has no prominent steamed bun peak. The crystallinity of the attapulgite specimen is high, and the amorphous substance is less.

From Figure 8b, we can see that, after adding lime or cement to the attapulgite-modified loss, two new peaks appear between 27.9° and 28.5°. According to previous studies, substances commonly produced are calcium silicate hydrate (CSH) and calcium aluminate hydrate (CAH) in this area [24]. After adding lime or cement, it reacts with active silicon oxide and alumina in loess and attapulgite to produce gelling products calcium silicate hydrate (CSH) and calcium aluminate hydrate (CAH). Additionally, calcium silicate hydrate (CSH) and calcium aluminate hydrate (CAH) effectively connect loess particles, thus reducing the permeability coefficient of modified loess.

**Figure 9:**
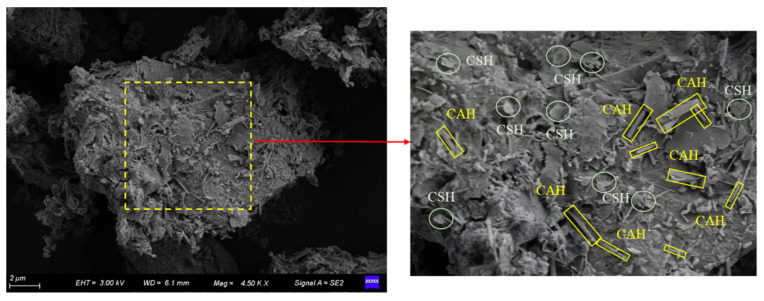
CAH and CSH in modified loess.

Figure 9 shows the CSH and CAH existence forms in modified loess. In this figure, we see that the shape of CSH is flaky while CAH is columnar [24]. Through comparing the EMS photos, it can be found that after adding lime and cement in attapulgite-modified loess, a new substance was formed in the shape of a rod. This material was not seen in the modified loess, attapulgite, and attapulgite loess. Combined with XRD analysis results, it can be determined that the substances are CSH and CAH. They are also the main component of cement. The generation of these substances made the modified soil denser and better integrated.

### 3.6. Shear Strength Test

**Figure 10:**
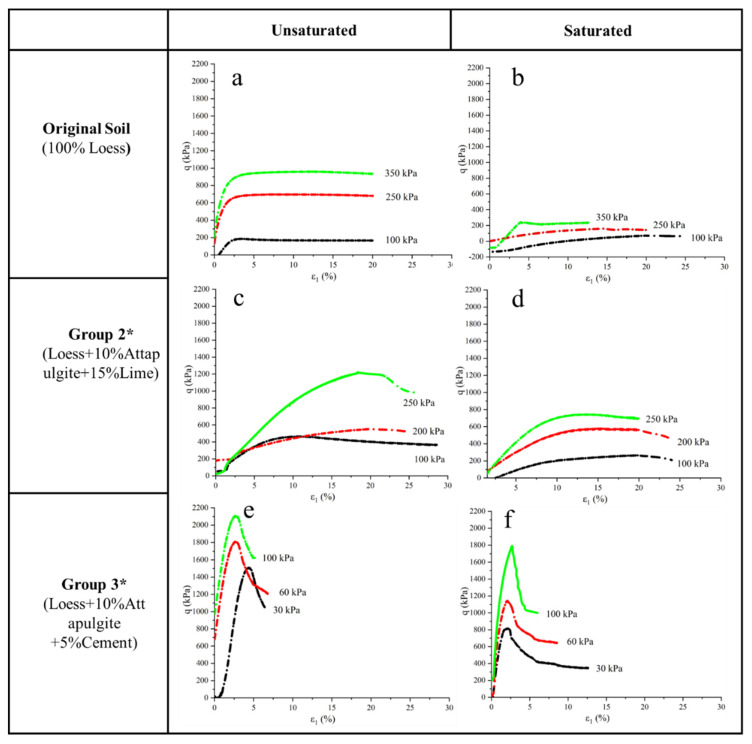
Stress–strain curve: (**a**) unsaturated loess; (**b**) saturated loess; (**c**) unsaturated attapulgite-lime-modified loess; (**d**) saturated attapulgite-lime-modified loess; (**e**) unsaturated attapulgite-cement-modified loess; (**f**) saturated attapulgite-cement-modified loess. * Optimum permeability coefficient.

Figure 10 shows the stress–strain curve of the consolidation drainage triaxial test. Since this test mainly studied the shear strength of modified loess, this curve shows the shear test results in the second stage. In contrast, the figure did not reflect consolidation drainage results in the first stage. Due to the different strengths of different specimens, the strain has occurred in consolidation and drainage in the first stage, so the starting point of part of the curve is not zero. Figure 10a,b were the test result of pure loess, Figure 10a was an unsaturated specimen, and Figure 10b was a saturated specimen. It can be seen from Figure 10a that with the increase in radial pressure, the stress increases with the same strain. In addition, under different radial pressures, the stress does not rise after reaching a specific value. However, the strain keeps increasing, and the curve is horizontal. As shown in Figure 10b, the stress of the saturated loess specimen is smaller than that of the unsaturated specimen under the same confining pressure. It also presents a curve close to horizontal when it reaches a specific value. It can be seen from Figure 10a,b that there is no apparent shear failure of saturated and unsaturated loess in the triaxial test. However, the axial strain keeps increasing, and the test ends when the strain reaches 20%.

Figure 10c,d shows the loess modified test results with attapulgite and lime. It can be seen from Figure 10c,d that the shear strength of the specimen is significantly improved when 10% attapulgite and 15% lime are added to the loess. Moreover, the shear strength of the unsaturated specimen in Figure 10c is more significant than that of the saturated specimen in Figure 10d under the same confining pressure. Unlike the results of pure loess specimens, a stress peak appeared in Figure 10c,d. The stress began to decline after reaching a specific value. With the decrease in stress, the strain continued to increase. Therefore, it can be determined that local failure occurred at the stress peak. After the failure, the strain continued to increase due to residual stress. Finally, the strain of the specimen reached 20%.

Figure 10e,f is the test result of attapulgite and cement modified loess. It can be seen from the figures that this group has the most significant stress among the three groups of experiments. Due to the equipment range, the confining pressure of this group is 30, 60, and 100 kPa, respectively. However, under the same confining pressure of 100 kPa, the maximum stress of this group of unsaturated specimens reached 2200 kPa, while the stress of the second and first groups was 440 and 200 kPa, respectively. It can also be seen from the figure that there is an obvious stress peak value in the experiment. After the stress reaches the maximum value, the stress drops sharply, and the strain increases continuously, similar to the experimental curve of brittle materials [25]. It can be concluded that shear failure occurred in the specimen in this experiment.

**Figure 11:**
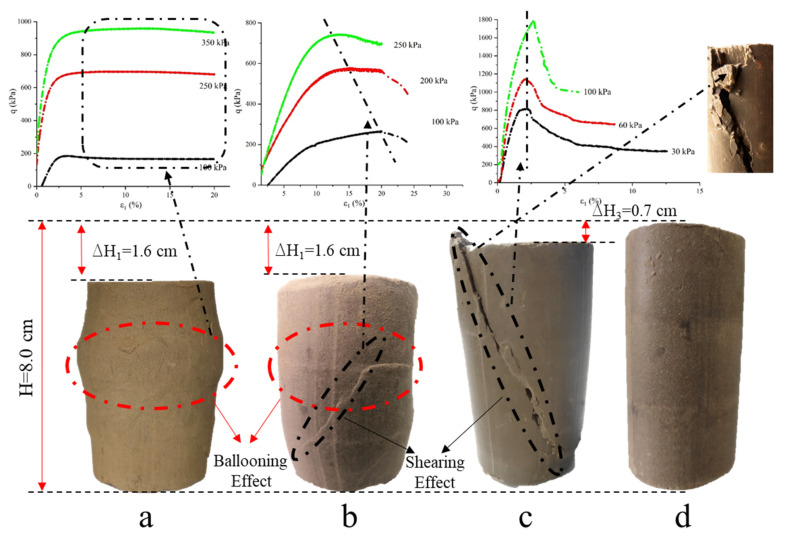
The failure mode of specimen in the shear test: (**a**) pure loess specimen; (**b**) attapulgite-lime-modified loess specimen; (**c**) attapulgite-cement-modified loess specimen; (**d**) original contrast specimen.

Figure 11 shows the failure mode of the specimen after the shear test. Figure 11a is the result of a pure loess specimen. It can be seen from the figure that the specimen does not undergo shear damage but shows an expansion effect. The middle diameter of the specimen becomes larger, and the height decreases due to axial stress. The deformation characteristics are consistent with results such as Figure 10a. The loess specimen is destroyed because strain continues to increase, eventually achieving the experimental value, and the experiment ends. Figure 11b shows the loess specimen modified by attapulgite and lime. It can be seen from figure that oblique cracks occur in the lower part of the specimen, which does not run through the whole specimen but only appears in the lower part. At the same time, the diameter of the middle region of the specimen increased. However, the diameter variation was not as significant as that of the pure loess specimen. It can also be seen in Figure 10c that the specimen strain was not very large when the stress peak occurred, and the stress continued to increase after the peak. However, the increase was not as significant as that of the pure loess specimen. Therefore, the shear failure pattern of the loess specimens modified by attapulgite and lime is consistent with the stress–strain curve.

Figure 11c is a specimen of the loess modified by attapulgite-cement. As seen from the secondary figure, obvious shear failure occurred in the specimen during the shear process, resulting in cracks from top to bottom. The crack angle is about 45 degrees, similar to the shear failure angle in Mohr Coulomb’s law. After shear failure, the right half of the specimen slips downward, resulting in significant strain. It can also be seen in Figure 10e that the stress rises rapidly before reaching the peak, and the strain change is small, only 2%. The stress decreases rapidly after the peak value and the strain increases. On the other side of the failure surface, it can be seen that the whole piece of the soil of the specimen falls off. Hence, the specimen’s strength is very high and has similar properties to brittle materials. Since this experiment mainly tested the maximum shear stress of the specimen, the experiment was artificially ended after the shear failure of the specimen. The strain did not reach 20%.

The above analysis shows that the saturated loess specimen with low strength mainly has a bulging effect in the shear test. Its diameter increases while its height decreases. With increased specimen strength, the failure mode of attapulgite, lime, and cement-modified loess specimens change from ballooning failure to shear failure.

**Figure 12:**
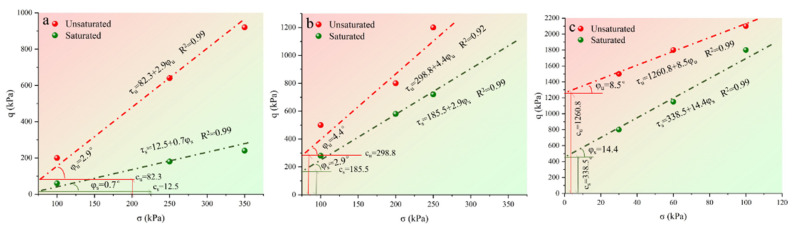
Shear strength envelope: (**a**) pure loess; (**b**) attapulgite-lime-modified loess; (**c**) attapulgite-cement-modified loess.

Figure 12 is the envelope of the shear strength of different specimens. Figure 12a is the envelope line of the loess specimen. It can be seen from the figure that the cohesion and friction angle of the unsaturated specimen are larger than the saturated specimen, and the cohesion of the unsaturated specimen is only 12.5 kPa. Figure 12b shows the envelope line of the attapulgite-lime-modified loess specimen. It can be seen from the figure that the cohesion and friction Angle of saturated and unsaturated modified loess both increase, and the value of the unsaturated specimen is larger than that of the saturated specimen. Figure 12c is the envelope line of the loess specimen modified by attapulgite-cement. As shown in Figure 12b, the value of friction angle cohesion is greatly improved after the modification of loess. The cohesion of the saturated specimen is 338.5 kPa, and that of the unsaturated specimen is 1260.8 kPa. Based on the previous microstructure and composition analysis, calcium aluminate hydrate and calcium silicate hydrate were formed in the modified loess. These substances made the modified loess stick together and created a structure with higher integrity. This process would increase the bite force between particles and increase cohesion and friction Angle. It can be concluded that the shear strength of the modified loess is significantly improved compared with the pure loess, among which the effects of attapulgite-cement-modified loess are the most significant [25].

## 4. Conclusions

This study aimed to investigate the feasibility of using modified loess soil as a landfill liner material and provide prerequisite support for studying the adsorption performance of the soil on landfill leachate. This research tested the seepage mechanism and shear strength of attapulgite-modified loess. SEM, XRD, and NMR were used to analyze the mechanism from the microscopic point of view. Finally, it yielded the following significant developments:

1. Attapulgite can significantly reduce the permeability coefficient of loess. The permeability coefficient dropped from 3.0 × 10^−6^ cm/s to 4.2 × 10^−7^ cm/s. However, the effect is not obvious when the content of attapulgite exceeds 10%. The permeability coefficient of modified loess can be further reduced by adding 15% lime or 5% cement based on 10% attapulgite content. The permeability coefficient can be reduced to 4.5 × 10^−8^ cm/s and 9.2 × 10^−8^ cm/s, respectively, which can meet the impermeability requirements of the landfill site. Through microscopic test and composition analysis, it is found that the anti-seepage mechanism of the loess modified by attapulgite is that attapulgite fills the pores between loess particles. The acicular attapulgite combines with loess to form a flake integral structure, thus reducing the permeability coefficient. When lime and cement are added to loess, calcium aluminate hydrate is created, which binds soil particles together to form a block structure with better overall integrity, reducing the permeability coefficient of the modified soil, among which attapulgite and cement modified loess have the best anti-seepage effect.

2. According to the static triaxial shear test, the shear strength of the loess modified with attapulgite-lime and the loess modified with attapulgite-cement is greatly improved compared with the pure loess cohesion. The friction angle of the specimen is also increased. The shear strength of the same specimen under unsaturated conditions is more significant than that under saturated conditions. Swelling failure mainly occurred in the loess specimens with low shear strength, and shear failure happened with increased shear strength.

This study shows that the loess modified with attapulgite-lime and the loess modified with attapulgite-cement can meet the impermeability requirements of the landfill lining materials and significantly improve its shear strength. The results indicated that the modified soil could be used as the lining material of landfills in loess areas. They provided the basis for further study on the adsorption performance of attapulgite to leachate in landfills. Attapulgite-modified loess is a brand new, ecological, environmentally friendly material. It can prevent seepage and absorb the heavy metal ions in the leachate. Finally, the new lining material will provide double security for the landfill and new impermeable material with higher safety and lower cost for the design and construction of landfill sites in the loess region. At the same time, since modified loess is a brittle material, its impermeability and stability under seismic loading in the actual application need further study. In addition, the impermeability of the modified soil under the action of wet and dry cycles in the application environment also needs to be studied specifically.

## 5. Possible Directions for Future Studies

This study shows that attapulgite-lime and the loess modified with attapulgite-cement can meet the impermeability requirements of landfills. In the next step, we will further study modified loess’s adsorption effect on landfills’ leachate, shear strength, and stability under the changing temperature, upper load, and seismic load.

## Figures and Tables

**Table 1:** The basic physical properties of loess and attapulgite.

Particle Size (mm)	Loess (%)	Attapulgite (%)
>1.1	0.37	0
1.1–0.25	19.43	0
0.25–0.075	23.8	0
0.075–0.05	9.9	4.6
0.05–0.01	28.4	67.72
0.01–0.005	9.1	22.7
<0.005	9.0	5.0
Liquid Limit, LL	28.7%	-
Plastic Limit, PL	14.9%	-
Plastic Index, PI	13.8	-

**Table 2:** The elemental chemical composition of loess and attapulgite.

Types of Chemical	Loess (%)	Attapulgite (%)
Strontium oxide, SrO	0.07	-
Iron (ii) oxide, Fe_2_O_3_	6.6	5.5
Silicon dioxide, SiO_2_	53.0	63.3
Aluminum oxide, Al_2_O_3_	15.6	11.6
Calcium oxide, CaO	1.95	1.14
Titanium dioxide, TiO_2_	0.93	0.84
Potassium oxide, K_2_O	3.16	1.07
Manganese oxide, MnO	0.13	15.58
Sodium oxide, Na_2_O	2.16	0.15
Magnesium oxide, MgO	12.8	11.35
Phosphorus pentoxide, P_2_O_5_	-	0.4

**Table 3:** Cement performance index.

Initial Jelling Time (min)	Final Setting Time (min)	3 Days		28 Days	
		Flexural Strength	Compressive Strength	Flexural Strength	Compressive Strength
>45	<600	2.5 MPa	10 MPa	5.5 MPa	32.5 MPa

**Table 4:** Loess modification mixtures composition divided into three main groups.

Group 1		Group 2			Group 3		
Loess (%)	Attapulgite (%)	Loess (%)	Attapulgite (%)	Lime (%)	Loess (%)	Attapulgite (%)	Cement (%)
98	2	87	10	3	87	10	3
96	4	83	10	7	85	10	5
94	6	80	10	10	83	10	7
92	8	78	10	12	80	10	10
90	10	75	10	15	78	10	12
84	16	70	10	20	75	10	15

**Table 5:** Optimum moisture content (OMC) and maximum dry density (MDD) results for the various percentage of materials (attapulgite, lime, and cement).

Percentage of Attapulgite (%)	Parameter	2	4	6	8	10	16
**Group 1** **(Loess + %Attapulgite)**	OMC ^1^ (%)	13.9	13.23	16.52	17.01	17.3	18.85
	MDD ^2^ (g/cm^3^)	1.85	1.84	1.74	1.71	1.70	1.69
Percentage of Lime (%)	Parameter	3	7	10	12	15	20
**Group 2 (Loess + 10%Attapulgite + %Lime**	OMC (%)	17.0	17.5	17.8	18.2	19.8	21.4
	MDD (g/cm^3^)	1.69	1.65	1.62	1.61	1.57	1.55
Percentage of Cement (%)	Parameter	3	5	7	10	12	15
**Group 3 (Loess + 10%Attapulgite + %Cement)**	OMC (%)	16.8	17.3	17.3	17.4	17.5	17.6
	MDD (g/cm^3^)	1.78	1.76	1.75	1.74	1.73	1.73

^1^ OMC—optimum moisture content; ^2^ MDD—maximum dry density.

## Data Availability

The data used to support the findings of this study are available from the corresponding author upon request.
